# Body Mass Index and the Risk of Dementia among Louisiana Low Income Diabetic Patients

**DOI:** 10.1371/journal.pone.0044537

**Published:** 2012-09-05

**Authors:** Gang Hu, Ronald Horswell, Yujie Wang, Wei Li, Jay Besse, Ke Xiao, Honglei Chen, Jeffrey N. Keller, Steven B. Heymsfield, Donna H. Ryan, Peter T. Katzmarzyk

**Affiliations:** 1 Pennington Biomedical Research Center, Baton Rouge, Louisiana, United States of America; 2 Louisiana State University Health Care Service Division, Baton Rouge, Louisiana, United States of America; 3 School of Human Ecology, Louisiana State University Agricultural Center, Baton Rouge, Louisiana, United States of America; 4 School of Public Health, Louisiana State University Health Science Center, New Orleans, Louisiana, United States of America; 5 Epidemiology Branch, National Institute of Environmental Health Sciences, Research Triangle Park, North Carolina, United States of America; Cardiff University, United Kingdom

## Abstract

**Background:**

The association between obesity and dementia risk remains debatable and no studies have assessed this association among diabetic patients. The aim of our study was to investigate the association between body mass index (BMI) and dementia risk among middle and low income diabetic patients.

**Methodology/Principal Findings:**

The sample included 44,660 diabetic patients (19,618 white and 25,042 African American) 30 to 96 years of age without a history of dementia in the Louisiana State University Hospital-Based Longitudinal Study. During a mean follow-up period of 3.9 years, 388 subjects developed incident dementia. The age- and sex-adjusted hazards ratios (HRs) for incident dementia at different levels of BMI (≤25, 25–26.9, 27–29.9, 30–34.9, and ≥35 kg/m^2^) were 1.00, 0.53 (95% CI 0.34–0.83), 0.29 (0.18–0.45), 0.37 (0.25–0.56), and 0.31 (0.21–0.48) (P_trend_<0.001) in white diabetic patients, and 1.00, 1.00 (95% CI 0.62–1.63), 0.62 (0.39–0.98), 0.56 (0.36–0.86), and 0.65 (0.43–1.01) (P_trend_ = 0.029) in African American diabetic patients. Further adjustment for other confounding factors affected the results only slightly. There was a significant interaction between race and BMI on dementia risk (χ^2^ = 5.52, 1df, p<0.025), such that the association was stronger in white patients. In stratified analyses, the multivariate-adjusted inverse association between BMI and risk of dementia was present in subjects aged 55–64 years, 65–74 years, and ≥75 years, in men and women, in non-smokers and smokers, and in subjects with different types of health insurance.

**Conclusions/Significance:**

Higher baseline BMI was associated with a lower risk of dementia among diabetic patients, and this association was stronger among white than among African American diabetic patients.

## Introduction

Obesity and diabetes are two important public health problems in the US [1], [2]. Two in three adults in the US are currently classified as overweight or obese (body mass index [BMI]≥25 kg/m^2^) and one-third of them are frankly obese (BMI≥30 kg/m^2^) [1]. Diabetes is considered “the epidemic of the 21^st^ century”, affecting approximately 24 million individuals in the US alone, or nearly 8% of the US population [2], [3]. Among US diabetic patients, the prevalence of overweight or obesity has increased up to 80% or more [4]. Obesity is associated with increased risks of cardiometabolic and neurological diseases, including hypertension [5], diabetes [6], coronary heart disease [7]–[9], heart failure [10], stroke [11] and Parkinson's disease [12]. Louisiana has one of the highest rates of adult obesity and diabetes in the US [13].

In recent years, prospective studies have assessed the association between obesity and the risk of dementia, but the results are inconsistent [14]–[16]. Several studies have suggested that obesity in midlife contributes significantly to the development of dementia [17]–[20]. However, in very old people, overweight or obesity is related to a lower risk of dementia compared with a normal weight [20]–[24]. All of these studies were focused on general population-based samples [16]–[24], however, no studies assess this association among diabetic patients although most diabetic patients are overweight or obese. Moreover, no studies assess if this association is race-specific although there is a race-specific prevalence of Alzheimer's disease/dementia [25]. In this study, we examined the association between BMI and the risk of dementia in a large Hospital-Based Longitudinal Study of African American and White diabetic patients within the Louisiana State University (LSU).

## Methods

### Study Population

LSU Health Care Services Division (LSUHCSD) operates seven public hospitals and affiliated clinics in Louisiana, which provide quality medical care to the residents of Louisiana regardless of their income or insurance coverage [26], [27]. Overall, LSUHCSD facilities have served about 1.6 million patients (35% of the Louisiana population) since 1990. In the whole population served by the LSUHCSD hospitals, about 46% of patients qualify for free care (by virtue of being low income and uninsured – any individual or family unit whose income is at or below 200% of Federal Poverty Level), about 10% of patients are self-pay (uninsured, but incomes not low enough to qualify for free care), about 20% of patients are covered by Medicaid, about 14% of patients have Medicare, and about 10% of patients are covered by commercial insurance [26], [27]. Administrative (name, address, date of birth, gender, race/ethnicity, types of insurance, family income, and smoking status), anthropometric (date of examination, measurements of body weight, height, and blood pressure for each visit), laboratory (test code, test collection date, test result values, and abnormal flag), clinical diagnosis (date of diagnosis, diagnosis code, priority assigned to diagnosis, International Classification of Disease Code [ICD]-9, and CPT procedure codes), and medication (medication generic name, pharmacopeia dispensable drug ID, medication strength-dose form, medication strength units, medication rote code and description, medication form, etc.) data collected at these facilities are available in electronic form for both inpatients and outpatients from 1999. Using these data, we have established the LSU Hospital-Based Longitudinal Study (LSUHLS) [26]. Longitudinal studies using the electronic dataset from medical records have been extensively used in the US and Europe [4], [28], [29]. A cohort of diabetic patients was identified by using the ICD-9 250 through the LSUHLS database between January 1, 1999, and June 1, 2009. LSUHCSD's internal diabetes disease management guidelines call for physician confirmation of diabetes diagnoses by applying the American Diabetes Association criteria: a fasting plasma glucose level ≥126 mg/dL; 2-hour glucose level ≥200 mg/dL after a 75-g 2-hour oral glucose tolerance test (OGTT); one or more classic symptoms plus a random plasma glucose level ≥200 mg/dL [26], [30], [31]. The first record date of body weight and height measurements among prior or currently diagnosed diabetic patients was used to establish the baseline for each patient in the present analyses due to the design of the cohort study. The present study included 44,660 diabetic patients (19,618 White and 25,042 African American) who were 30 to 96 years of age without a history of dementia, and with complete data on any required variables. Compared with diabetic patients excluded in the present analyses due to missing data on any required variables, the diabetic patients included in the present analyses were younger (53.5 vs. 55.8 years old), had less frequency of African Americans (56.1% vs. 59.3%), and less males (38.4% vs. 45.5%). The study and analysis plan were approved by both the Pennington Biomedical Research Center and LSU Health Sciences Center Institutional Review Boards, LSU System. We do not obtain informed consent from all participants involved in our study because we use the electronic dataset from medical records.

### Baseline measurements

The patient's characteristics, including age of diabetes diagnosis, types of insurance, gender, race/ethnicity, family income, smoking status, body weight, height, BMI, blood pressure, total cholesterol, high-density lipoprotein (HDL) cholesterol, low-density lipoprotein (LDL) cholesterol, triglycerides, HbA_1c_, estimated glomerular filtration rate (eGFR), and medication (antihypertensive drug, cholesterol lowing drug and antidiabetic drug) were extracted from the computerized hospitalization records.

### Prospective follow-up

Follow-up information was obtained from the LSUHLS inpatient and outpatient database by using the unique number assigned to every patient who visits the LSUHCSD hospitals each time. The mean times of visiting hospitals during the follow-up period for each patient were 12.9. The diagnosis of dementia was the primary endpoint of interest of the study, and was defined according to the following ICD-9: Alzheimer disease 331.0; vascular dementia 290.4; and other dementias 331.1, 331.2, 331.7, 331.82, 290.0, 290.1, 290.10, 290.11, 290.12, 290.13, 290.20, 290.21, 290.3, 290.80, and 290.90. Dementia classification was completed by consensus of neurologists and/or psychiatrists by using standardized and established neuropsychiatric tests. LSUHCSD's consensus committee determined the presence of dementia and its subtypes based on the Diagnostic and Statistical Manual of Mental Disorders, Fourth Edition, criteria for dementia [32], the National Institute of Neurological and Communicative Disorders and Related Disorders Association criteria for Alzheimer disease [33], and the NINDS-AIREN criteria for vascular dementia [34]. Follow-up of each cohort member continued until the date of the diagnosis of dementia, the date of the last visit if the subject stopped use of LSUHCSD hospitals, or June 30, 2010.

### Statistical analyses

Race-specific differences in risk factors based on different levels of BMI were tested using analysis of variance or logistic regression after adjustment for age and sex. The association between BMI at baseline and the risk of dementia was analyzed by using Cox proportional hazards models. BMI was evaluated in the following 2 ways: (1) as 5 weight categories (<25 [reference group], 25–26.9, 27–29.9, 30–34.9, and ≥35 kg/m^2^), and (2) as a continuous variable. Different levels of BMI were included in the models as dummy and categorical variables, and the significance of the trend over different categories of BMI was tested in the same models by giving an ordinal numeric value for each dummy variable. The proportional hazards assumption in the Cox model was assessed with graphical methods, and with models including time-by-covariate interactions.[35] In general, all proportionality assumptions were appropriate. All analyses were adjusted for age and sex (the age- and sex-adjusted model) and further for smoking, income, systolic blood pressure, diabetes type, duration of diabetes, HbA1c, LDL cholesterol, triglycerides, eGFR, use of antihypertensive drugs, use of diabetes medications, and use of cholesterol-lowering agents (the multivariate-adjusted model). Since the interactions between sex and BMI on the risk of dementia were not statistically significant among both white and African Americans, data for men and women were combined in some analyses. To avoid the potential bias due to possible early weight loss during the subclinical stage prior to the diagnosis of dementia [36], additional analyses were carried out excluding the subjects who were diagnosed with dementia during the first two years of follow-up. Statistical significance was considered to be P<0.05. All statistical analyses were performed with PASW for Windows, version 20.0 (IBM SPSS Inc, Chicago, III).

## Results

General characteristics of the study population at baseline are presented in Table 1. Patients who developed dementia during follow-up were older, their BMI and baseline serum total cholesterol and triglycerides were lower, and they had higher family income, were less current smokers, more often chronic kidney disease, and less using glucose-lowering medicine compared with those who remained free of dementia (Table 1).

**Table 1:** General characteristics among patients of diabetes by the outcome during follow-up.

	African American		P value	White American		P value
	No dementia	Dementia		No dementia	Dementia	
No. of participants	24 854	188		19418	200	
Age (mean, yr)	52.6	70.5	<0.001	43.3	72.0	<0.001
Income (mean, $/family)	10 279	19 620	0.006	8303	15 333	0.013
Body mass index (mean, kg/m^2^)	33.7	32.6	0.049	34.3	30.7	<0.001
Systolic blood pressure (mean, mmHg)	146	143	0.16	141	137	0.026
Diastolic blood pressure (mean, mmHg)	82	81	0.27	77	76	0.17
Total cholesterol (mean, mg/dL)	185	178	0.025	188	180	0.012
High-density lipoprotein cholesterol (mean, mg/dL)	46	46	0.88	42	41	0.31
Low-density lipoprotein cholesterol (mean, mg/dL)	114	115	0.73	111	110	0.67
Triglycerides (mean, mg/dL)	109	101	0.039	137	128	0.033
HbA1c (mean, %)	7.87	7.55	0.13	7.16	7.05	0.47
Duration of diabetes (mean, yr)	2.85	3.04	0.42	2.28	2.10	0.41
Prevalence of current smoking (%)	33.5	19.8	<0.001	38.6	18.9	<0.001
Types of insurance			<0.001			<0.001
Free Care	54.4	30.8		58.1	27.5	
Self-pay	15.5	8.0		15.7	8.5	
Medicare	5.3	34.6		8.6	49.5	
Medicaid	12.3	14.9		6.7	4.0	
Commercial insurance	12.5	11.7		10.9	10.5	
Types of diabetes			0.32			0.58
Type 1	0.6	1.1		0.7	0.5	
Type 2	99.4	98.9		99.3	99.5	
GFR (mL/min/1.73m^2^) (%)			<0.001			<0.001
≥90	52.5	26.6		34.6	16.2	
60–89	36.3	50.5		49.0	46.0	
30–59	9.3	21.3		14.8	34.3	
15–29	1.2	1.1		1.2	2.5	
<15	0.8	0.5		0.4	1.0	
Uses of medications (%)						
Antihypertensive medications	83.6	88.2	0.17	77.5	69.0	0.031
Glucose-lowering medication	76.2	58.1	<0.001	75.2	63.8	0.003
Lipid-lowering medication	58.6	66.2	0.081	69.2	79.3	0.022

Data shown are means or percentages. All date except age adjusted for age and sex.

With increasing BMI, mean values of blood pressure, total and LDL cholesterol, triglycerides, and the prevalence of using antihypertensive drugs, diabetes medications, and cholesterol-lowing agents increased, while means in HDL cholesterol, family income, and the prevalence of current smoking decreased (Table 1). During a mean follow-up period of 3.9 years, 388 subjects (200 white and 188 African American) developed incident dementia.

The age- and sex-adjusted hazards ratios (HRs) for incident dementia at different levels of BMI (≤25, 25–26.9, 27–29.9, 30–34.9, and ≥35 kg/m^2^) were 1.00, 0.53 (95% CI 0.34–0.83), 0.29 (95% CI 0.18–0.45), 0.37 (95% CI 0.25–0.56), and 0.31 (95% CI 0.21–0.48) (P_trend_<0.001) in white diabetic patients, and 1.00, 1.00 (95% CI 0.62–1.63), 0.62 (95% CI 0.39–0.98), 0.56 (95% CI 0.36–0.86), and 0.65 (95% CI 0.43–1.01) (P_trend_ = 0.029) in African American diabetic patients, and 1.00, 0.71 (95% CI 0.51–0.98), 0.41 (95% CI 0.29–0.57), 0.45 (95% CI 0.34–0.60), and 0.44 (0.33–0.60) (P_trend_<0.001) in white and African American diabetic patients combined (adjusted also for race) (Table 2). After further adjustment for other confounding factors (smoking, income, systolic blood pressure, diabetes type, duration of diabetes, HbA1c, LDL cholesterol, triglycerides, eGFR, use of antihypertensive drugs, use of diabetes medications, and use of cholesterol-lowering agents), this inverse association remained significant among white, African American and the combined sample of diabetic patients (all P_trend_<0.05).

**Table 2:** Hazard ratio of dementia according to different levels of body mass index or body mass index as a continuous variable among diabetic patients.

	Body mass index (kg/m)					P for trend	One unit increase (continuous variable)
	<25	25.0–26.9	27–29.9	30–34.9	≥35		
African American	3301	2029	3869	6225	9618		
No. of cases	51	25	28	38	46		
Person-years	12,411	7,772	15,167	24,693	38,177		
Age and sex adjustment HR (95% CI)	1.00	1.00 (0.62–1.63)	0.62 (0.39–0.98)	0.56 (0.36–0.86)	0.65 (0.43–1.01)	0.029	0.98 (0.96–0.99)^b^
Multivariable adjustment HR (95% CI)^a^	1.00	0.95 (0.56–1.61)	0.60 (0.37–0.99)	0.54 (0.34–0.85)	0.68 (0.43–1.07)	0.048	0.98 (0.96–1.00)
White	2289	1490	2879	4851	8109		
No. of cases	76	25	23	39	37		
Person-years	8,459	5623	10,999	18,808	31,047		
Age and sex adjustment HR (95% CI)	1.00	0.53 (0.34–0.83)	0.29 (0.18–0.45)	0.37 (0.25–0.56)	0.31 (0.21–0.48)	<0.001	0.94 (0.91–0.96)^b^
Multivariable adjustment HR (95% CI)^a^	1.00	0.53 (0.33–0.84)	0.27 (0.16–0.45)	0.35 (0.23–0.54)	0.30 (0.19–0.47)	<0.001	0.94 (0.91–0.96)
Total c	5590	3519	6748	11 076	17 727		
No. of cases	127	50	51	77	83		
Person-years	20,871	13,395	26,166	43,501	69,224		
Age, sex and race adjustment HR (95% CI)	1.00	0.71 (0.51–0.98)	0.41 (0.29–0.57)	0.45 (0.34–0.60)	0.44 (0.33–0.60)	<0.001	0.96 (0.94–0.97)
Multivariable adjustment HR (95% CI)^a^	1.00	0.68 (0.48–0.96)	0.40 (0.28–0.56)	0.43 (0.31–0.58)	0.44 (0.32–0.61)	<0.001	0.96 (0.94–0.97)

a Adjusted for age, sex, race, income, types of insurance, smoking, systolic blood pressure, diabetes type, HbA1c, low-density lipoprotein cholesterol, GFR, use of antihypertensive drugs, use of diabetes medications, and use of cholesterol-lowing agents.

b χ^2^ = 5.52, 1df, p for interaction <0.025.

c Adjusted for race also.

When BMI was examined as a continuous variable, the age- and sex-adjusted HRs for each 1-unit increase in BMI were 0.94 (95% CI 0.92–0.96) in white diabetic patients, 0.98 (95% CI 0.96–0.996) in African American diabetic patients, and 0.96 (95% CI 0.94–0.97) in white and African American diabetic patients combined (adjusted also for race). There was a significant interaction between race and BMI on dementia risk (χ^2^ = 5.52, 1df, p<0.025), which indicated that the inverse association was stronger in white patients than in African American patients. After further adjustment for other confounding factors, this inverse association remained significant in white and whole diabetic patients and was almost significant among African American diabetic patients (HR 0.98, 95% CI 0.96–1.002).

After exclusion of participants who were diagnosed with dementia during the first two years of follow-up (n = 165), the multivariable-adjusted HRs for each 1-unit increase in BMI were 0.93 (95% CI 0.90–0.96) in white diabetic patients, 0.97 (95% CI 0.94–0.99) in African American diabetic patients, and 0.95 (95% CI 0.93–0.97) in white and African American diabetic patients combined (adjusted also for race) (data not shown).

In stratified analyses, the multivariate-adjusted inverse association between BMI and risk of dementia was present in subjects aged 55 to 64 years, 65–74 years, and 75 or more years, in men and women, in non-smokers and smokers, in subjects with different family income, and different types of health insurance (Table 3). In stratified analyses, we also found the multivariate-adjusted inverse association between BMI and the risk of Alzheimer disease, and between BMI and the risk of vascular dementia.

**Table 3:** Hazard ratio of dementia according to different levels of body mass among various subpopulations of diabetic patients.

	Body mass index (kg/m)					P for trend	P for interaction
	<25	25.0–26.9	27–29.9	30–34.9	≥35		
Age at baseline (years)							>0.5
30–54	1.00	1.64 (0.50–5.42)	0.74 (0.21–2.68)	0.25 (0.05–1.24)	0.66 (0.22–2.00)	0.22	
55–64	1.00	0.52 (0.23–1.18)	0.40 (0.20–0.81)	0.27 (0.13–0.53)	0.36 (0.20–0.65)	0.001	
65–74	1.00	0.64 (0.34–1.24)	0.43 (0.23–0.79)	0.48 (0.28–0.82)	0.43 (0.25–0.76)	0.018	
≥75	1.00	0.61 (0.38–0.98)	0.35 (0.20–0.59)	0.55 (0.36–0.86)	0.50 (0.29–0.84)	<0.001	
Sex							>0.1
Men	1.00	0.65 (0.40–1.07)	0.22 (0.12–0.41)	0.29 (0.17–0.50)	0.27 (0.15–0.49)	<0.001	
Women	1.00	0.68 (0.44–1.06)	0.56 (0.38–0.84)	0.54 (0.37–0.78)	0.53 (0.37–0.76)	0.003	
Smoking							>0.25
Never or even	1.00	0.58 (0.39–0.87)	0.42 (0.29–0.60)	0.42 (0.30–0.58)	0.44 (0.31–0.62)	<0.001	
Current	1.00	1.08 (0.57–2.05)	0.34 (0.15–0.75)	0.42 (0.21–0.85)	0.35 (0.17–0.73)	0.002	
Income ($/family)							>0.1
<1500	1.00	0.82 (0.47–1.42)	0.35 (0.19–0.64)	0.54 (0.34–0.88)	0.61 (0.38–0.97)	0.006	
≥1500	1.00	0.47 (0.22–0.99)	0.29 (0.14–0.58)	0.24 (0.12–0.47)	0.35 (0.19–0.65)	<0.001	
Type of insurance							>0.25
Free Care	1.00	0.72 (0.36–1.45)	0.50 (0.27–0.95)	0.40 (0.22–0.73)	0.45 (0.26–0.80)	0.022	
Self-pay	1.00	0.32 (0.09–1.17)	0.13 (0.03–0.58)	0.22 (0.08–0.60)	0.20 (0.08–0.54)	0.002	
Medicare, Medicaid, and commercial insurance	1.00	0.72 (0.48–1.10)	0.40 (0.25–0.62)	0.50 (0.34–0.74)	0.51 (0.34–0.78)	<0.001	
Types of diabetes							
Type 1	-	-	-	-	-	-	
Type 2	1.00	0.66 (0.47–0.94)	0.40 (0.28–0.56)	0.43 (0.32–0.59)	0.45 (0.33–0.62)	<0.001	
Incident outcomes							
Alzheimer disease	1.00	0.59 (0.38–0.90)	0.45 (0.30–0.66)	0.45 (0.31–0.65)	0.27 (0.25–0.54)	<0.001	
Vascular dementia	1.00	0.82 (0.30–2.22)	0.15 (0.03–0.70)	0.15 (0.04–0.54)	0.39 (0.15–0.98)	0.009	
Other dementia	1.00	1.11 (0.53–2.33)	0.39 (0.16–0.94)	0.57 (0.28–1.19)	0.94 (0.47–1.87)	0.10	

Adjusted for age, sex, race, income, types of insurance, smoking, systolic blood pressure, diabetes type, HbA1c, low-density lipoprotein cholesterol, use of antihypertensive drugs, use of diabetes medications, and use of cholesterol-lowing agents.

In addition, we have done an additional analysis by using the age- and race-specific quartiles of BMI. The multivariable-adjusted HRs for incident dementia across age- and race-specific quartiles of BMI were 1.00, 0.48 (95% CI 0.32–0.70), 0.36 (95% CI 0.24–0.56), and 0.47 (95% CI 0.31–0.71) (P_trend_<0.001) in white diabetic patients, and 1.00, 0.52 (95% CI 0.34–0.81), 0.57 (95% CI 0.38–0.87), and 0.72 (95% CI 0.48–1.08) (P_trend_ = 0.011) in African American diabetic patients, and 1.00, 0.50 (95% CI 0.37–0.66), 0.46 (95% CI 0.34–0.62), and 0.58 (0.44–0.78) (P_trend_<0.001) in white and African American diabetic patients combined (adjusted also for race). In stratified analyses, the multivariate-adjusted inverse association between age- and race-specific quartiles of BMI and risk of dementia was present in subjects aged less than 60 years (P_trend_ = 0.028) (we combined age groups of 30–39 years, 40–49 years, and 50–59 years as one group due to the very low incident cases of dementia in each age group), 60–69 years (P_trend_ = 0.005), 70–79 years (P_trend_ = 0.001), and 80 or more years (P_trend_ = 0.001).

## Discussion

To the best of our knowledge, this is the largest prospective analysis on BMI and dementia among diabetic patients. We found an inverse association between baseline BMI and the risk of dementia, and this association was stronger among white than among African American diabetic patients. In addition, we found that this inverse association was present in subjects aged 55 to 64 years, 65–74 years, and 75 or more years, in non-smokers and smokers, and in subjects with different types of health insurance.

Alzheimer's disease/dementia is the fifth leading cause of death in older US adults aged ≥65 years [25]. Recent studies have provided strong evidence for an important role of environmental factors in the etiology of dementia [37]. Several studies have suggested that the association between obesity and the risk of dementia may be age-dependent. High BMI in midlife has been found to be associated with an increased risk of dementia [17]–[20]; on the other hand, most studies on late life BMI and dementia found an inverse association [20]–[24], with only one exception [38]. In the present study, we found that high BMI was associated with a decreased risk of dementia among diabetic patients, and this significant inverse association was present in subjects aged 55 to 64 years, 65–74 years, and 75 or more years; a similar association was also observed among younger participants aged 30–54 years (midlife), although the statistical test was not significant due to the small number of dementia cases in this group (n = 26).

Our study was different from other general population-based studies [17]–[24]. All participants were low income diabetic patients: 55.9% of diabetic patients quality for free care, 15.5% of patients are self-pay, and 28.6% of patients are covered by Medicaid (9.8%), Medicare (7.1%), and Commercial insurance (11.7%); and most of patients were either overweight (27%) or obese (64.5%). Since diabetes has been found as one important risk factor for dementia [39], our study sample with diabetes was at high risk for dementia to begin with, and the design largely removed the potential impact of diabetes with dementia risk among the general population. This may also explain the finding that both high midlife and later life BMI were associated with a decreased risk of dementia in our study. In addition, the low income levels and a high proportion of free care among our study participants might also decrease or remove the potential impact of socioeconomic factors with both BMI and the risk of dementia.

Weight loss has been shown to be more common with co-morbidities at older ages and is often reflective of poor health. Several studies have indicated that early weight loss during the subclinical stage may precede the diagnosis of dementia [21]–[23]. Our study population is enriched with overweight and obese adults who are more likely to lose their weight compared with the general population [17]–[24]. This may contribute to the inverse association between baseline BMI and dementia risk in the next several years. Nevertheless, we carried out sensitivity analyses excluding participants who were diagnosed with dementia during the first two years of follow-up (n = 165), which can reduce the possibility of potential bias due to possible early weight loss during the subclinical stage prior to the diagnosis of dementia.

The present study is, to our knowledge, the first large prospective study to determine that the inverse association between baseline BMI and dementia risk was stronger among white than among African American diabetic patients. Racial differences in abdominal depot-specific adiposity may partly explain the above race-specific association. Abdominal subcutaneous adipose tissue has been observed to be higher in African American men and women compared with white men and women [40], [41]. Several studies have suggested that increased amounts of subcutaneous fat may be associated with decreased risk of mild cognitive impairment [42]. High abdominal subcutaneous adipose tissue in African Americans may eliminate the effect of BMI on dementia risk compared with white Americans. Other studies with abdominal depot-specific adiposity measurement are further needed to assess this question between white and African Americans.

Potential explanations of this association among diabetic patients are unclear. As explained above, weight loss immediately before dementia identification may contribute to, but not entirely explain this association. Finally, it is also likely that obesity may indeed relate to a lower risk of dementia among diabetic patients. It has been shown that high BMI may be associated with increased levels of insulin-growth factor I [43], and leptin hormone [44]. Several studies have indicated that higher levels of insulin-growth factor I may be associated with better general cognition [45] and serum leptin levels were inversely associated with the risk of Alzhemier disease [46].

There are several strengths in our study, including the large sample size, high proportion of African Americans, and the use of administrative databases to avoid differential recall bias. In addition, participants in this study use the same public health care system which minimizes the influence from the accessibility of health care, particularly in comparing African Americans and Whites. One limitation of our study is that our analysis was not performed on a representative sample of the population which limits the generalizability of this study; however, LSUHCSD hospitals are public hospitals and cover over 1.6 million patients most of whom were middle or low income persons in Louisiana. The results of the present study will have wide applicability for the population with low income and without health insurance in the US. Second, we did not have data on other obesity indicators, such as waist and hip circumference and skin fold thickness, or data on the changes of BMI during the follow-up for all study samples. Third, the different survival time could have biased the results of the study because diabetic patients who were overweight or obesity in mid-life might have several other chronic diseases, such as hypertension [5], dyslipidemia [11], coronary heart disease [9], stroke [11], heart failure [10], and then died before they reached an older age. Thus the surviving overweight or obese diabetic older patients might be unusually healthy compared with the normal weight diabetic patients. Fourth, the relatively short follow-up period may increase the potential for reverse causality to explain the findings. Fifth, ascertainment of dementia was based on the hospital discharge register, rather than the standardized neurological assessments administered periodically to all cohort members. Thus less severe cases of dementia and/or mild cognitive impairment could not be identified, and it should not cause biased results but may only weaken the observed association. The method of using the hospital discharge register to diagnose dementia has been used in American and European cohort studies [17]–[19], [24], [47], [48]. The validity of dementia diagnosis by using hospital discharge register in these cohort studies was high [47]. Sixth, even though our analyses were adjusted for an extensive set of confounding factors, residual confounding due to the measurement error in the assessment of confounding factors, unmeasured factors such as physical activity, education, dietary factors, cognitive function for all patients, cannot be excluded.

In summary, in this large hospital-based cohort study, we found that higher baseline BMI was associated with a lower risk of dementia among diabetic patients, and this association was stronger among white than among African American diabetic patients.
